# Remodeling of the Lymph Node High Endothelial Venules Reflects Tumor Invasiveness in Breast Cancer and is Associated with Dysregulation of Perivascular Stromal Cells

**DOI:** 10.3390/cancers13020211

**Published:** 2021-01-08

**Authors:** Tove Bekkhus, Teemu Martikainen, Anna Olofsson, Mathias Franzén Boger, Daniel Vasiliu Bacovia, Fredrik Wärnberg, Maria H. Ulvmar

**Affiliations:** 1 The Beijer Laboratory, Department Immunology, Genetics and Pathology, Rudbeck Laboratory, Dag Hammarskjölds väg 20, Uppsala University, 75185 Uppsala, Sweden; tove.bekkhus@igp.uu.se (T.B.); Teemu.Martikainen.3903@student.uu.se (T.M.); anna.olofsson@ki.se (A.O.); mathias.franzen.boger@ki.se (M.F.B.); daniel.vasiliu.bacovia@akademiska.se (D.V.B.); 2 Department of Surgery, Institute of Clinical Sciences, Sahlgrenska Academy at the University of Gothenburg, 41345 Gothenburg, Sweden; fredrik.warnberg@gu.se

**Keywords:** lymph node, pre-metastatic changes, CCL21, high endothelial venule (HEV), T-lymphocytes, heparan sulfate, fibroblastic reticular cell (FRC), breast cancer, metastasis

## Abstract

**Simple Summary:**

Tumor draining lymph nodes (TDLNs) are the most common metastatic sites in human cancer but are also essential sites for induction of tumor immunity. How different types of primary tumors affect the anti-tumor immune response in the LNs is not fully understood. By analyzing biobank tissue from breast cancer patients, we demonstrate that invasive breast cancer induce dramatic pre-metastatic LN changes affecting the structure and function of the specialized LN vasculature and associated stromal cells, required for recruitment of T-lymphocytes into the LNs. These changes could not be seen in patients with non-invasive breast cancer and provide new insights of how invasive tumors can disrupt essential functions within the immune system. The data also shows promise of LN stromal and vascular changes as possible future biomarkers for prediction of disease progression in human cancer.

**Abstract:**

The tumor-draining lymph nodes (TDLNs) are primary sites for induction of tumor immunity. They are also common sites of metastasis, suggesting that tumor-induced mechanisms can subvert anti-tumor immune responses and promote metastatic seeding. The high endothelial venules (HEVs) together with CCL21-expressing fibroblastic reticular cells (FRCs) are essential for lymphocyte recruitment into the LNs. We established multicolor antibody panels for evaluation of HEVs and FRCs in TDLNs from breast cancer (BC) patients. Our data show that patients with invasive BC display extensive structural and molecular remodeling of the HEVs, including vessel dilation, thinning of the endothelium and discontinuous expression of the HEV-marker PNAd. Remodeling of the HEVs was associated with dysregulation of CCL21 in perivascular FRCs and with accumulation of CCL21-saturated lymphocytes, which we link to loss of CCL21-binding heparan sulfate in FRCs. These changes were rare or absent in LNs from patients with non-invasive BC and cancer-free organ donors and were observed independent of nodal metastasis. Thus, pre-metastatic dysregulation of core stromal and vascular functions within TDLNs reflect the primary tumor invasiveness in BC. This adds to the understanding of cancer-induced perturbation of the immune response and opens for prospects of vascular and stromal changes in TDLNs as potential biomarkers.

## 1. Introduction

Local and regional lymph nodes (LNs) are the most common metastatic sites in a large number of human cancer forms, including breast cancer (BC) [1]. In estrogen receptor (ER) positive invasive ductal carcinoma (IDC) LN metastasis is an independent prognostic factor that correlates with worse prognosis and risk for further metastatic spread [2,3]. Although experimental studies support that the LNs can act as a platform from which further metastatic spread occurs [4,5], tumor clonal studies by whole exon sequencing of metastatic BC does not support that metastatic seeding from axillary LNs is common [5]. However, another contributing factor to the link between tumor progression and LN metastasis in human cancer may be dysregulation of adaptive immunity. The LNs are essential both for induction of effective adaptive immune responses and maintenance of tolerance [6,7]. Immune cell access to and exit from the LN as well as immune regulation within the LN are hence limiting factors in anti-tumor immunity [8].

Both the mesenchymal LN stromal cells and the blood and lymphatic vasculature in the LN show unique specialization that is essential for the LN immune functions [6,7]. The LN specialized venules, known as high endothelial venules (HEVs), are built up by characteristic cuboidal shaped endothelial cells (ECs) and express sulfated carbohydrate ligands, including peripheral node addressin (PNAd) [9]. PNAd acts as a ligand for L-selectin/CD62L on lymphocytes, allowing the lymphocytes to tether and roll along the HEV endothelium [9]. Together with vessel-lining fibroblastic reticular cells (FRCs), which constitutively express the CCR7-ligands CCL19 and CCL21, HEVs provide essential units for recruitment of CD62L^+^/CCR7^+^ naïve T-and B-lymphocytes into the LN [10], an essential step for any adaptive immune response. Vascular changes in the TDLNs that affect HEV or FRC functions would thus be predicted to also impair the possibility to develop tumor immunity.

While it is well established that tumor-induced vascular changes in the primary tumor contribute to the tumor immune profile and metastatic progression [11], there is still limited insight into the potential pre-metastatic and metastatic vascular changes in TDLNs in human cancer. Current data is based on few patients without defined tumor types and with inconclusive results concerning the impact of LN metastasis [12,13]. Differences between non-invasive and invasive cancers are also unknown and the possible connections between vascular and other stromal changes and how this relates to changes in immune cells have not been evaluated. In this study, we address these questions by analyzing axillary LNs from BC patients with non-invasive ductal carcinoma in situ (DCIS) and LNs from patients with invasive ductal carcinoma (IDC), with and without LN metastasis. LNs from cancer-free organ donors (ODs) are used as controls.

## 2. Results

### 2.1. HEV Remodeling in the Paracortex (T-Cell Zone) Is Associated with Tumor Invasiveness But Is Independent of Nodal Metastasis

To allow the evaluation of HEV remodeling in formalin-fixed paraffin-embedded (FFPE) biobank samples, we established a multicolor antibody panel for simultaneous evaluation of HEVs (marked by expression of the vascular addressin PNAd and the broadly expressed tight junction protein Claudin-5), FRCs (marked by expression of the chemokine CCL21), and tumor cells (marked by cytokeratin). LNs from cancer-free ODs and axillary LNs from patients with DCIS, IDC with (IDC^met+^) and without LN metastasis (IDC^met−^) were stained. To be able to differentiate pre-metastatic and metastatic changes from potential tumor-type specific differences in patients with IDC, we selected groups of patients with the same basic primary tumor characteristics: luminal, estrogen receptor (ER) positive, human epidermal growth factor receptor 2 (HER2) negative, with and without LN metastasis (Table S1).

HEVs are mainly located in the paracortex (T-cell zone) and are absent in the cortex (B-cell zone) of the LNs [14]. Thus, HEV density will be strongly affected by the histological area chosen for analysis. Hence, for evaluating HEV density between patient groups in a comparable way we limited our initial analysis to HEVs in the paracortex, which can be defined by the presence of CCL21^+^ FRCs [15] (Figure S1). To define paracortex based on CCL21, rather than tissue morphology, exclude the risk to include non-paracortical areas or potential areas with complete loss of paracortical function and identity. We also excluded areas near tumor cells in metastatic LNs or near adipocytes (>200 µm from the tumor border or from parenchymal fat).

**Figure 1:**
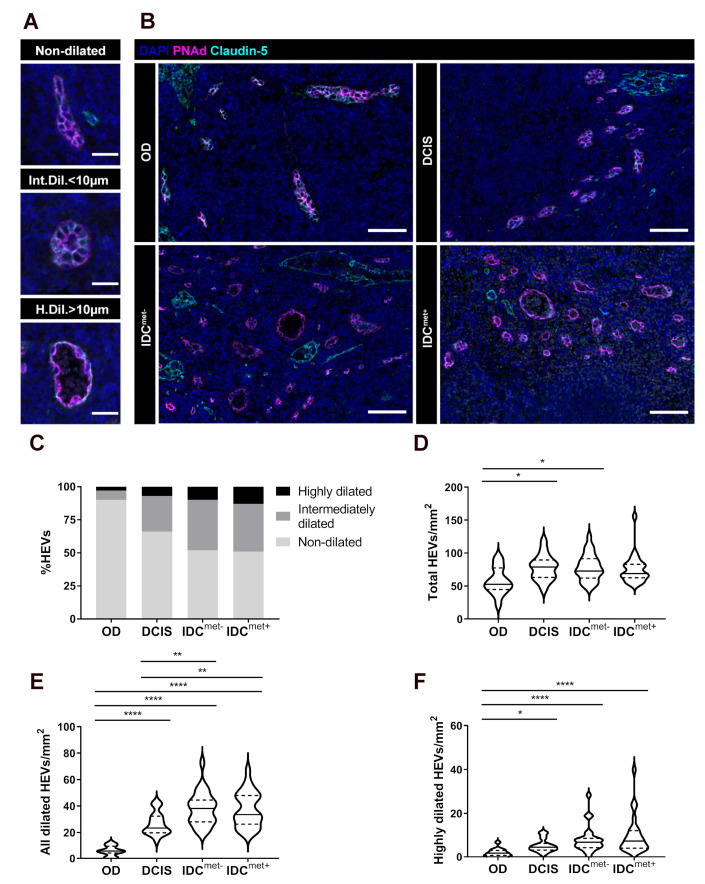
Paracortical HEV remodeling is associated with tumor invasiveness but is independent of LN metastasis. (**A**) Example images of a non-dilated (closed lumen), intermediately dilated (Int.Dil.) (lumen diameter <10 µm) and highly dilated (H.Dil.) (lumen diameter >10 µm) HEV. Scale bar: 25 µm. (**B**) Representative images of HEVs, (PNAd^+^/Claudin-5^+^) in organ donors (OD), non-invasive DCIS, and invasive IDC without (IDC^met−^) and with (IDC^met+^) LN metastasis. Scale bar 75 µm. (**C**) Overview of the fraction of highly dilated, intermediate dilated, non-dilated HEVs of all HEVs in each patient group. (**D–F**) HEV numbers normalized to the paracortical area (HEVs/mm^2^); (**D**) Total number of HEVs, (**E**) Number of all dilated HEVs (intermediately and highly dilated) and (**F**) Number of highly dilated HEVs. (**D–F**) Solid line indicate median and dotted lines quartiles. n = 15/10 OD, n = 19/19 DCIS, n = 25/25 IDC^met−^, n = 25/24 IDC^met+^ LNs/patients. Ordinary ANOVA and Holm-Sidak’s multiple comparison test were used in (**C**) and Kruscal Wallis and Dunn’s multiple comparisons tests were used for statistical analysis in (**B**) and (**D**). * *p* < 0.05, ** *p* < 0.01, **** *p* < 0.0001.

**Figure 2:**
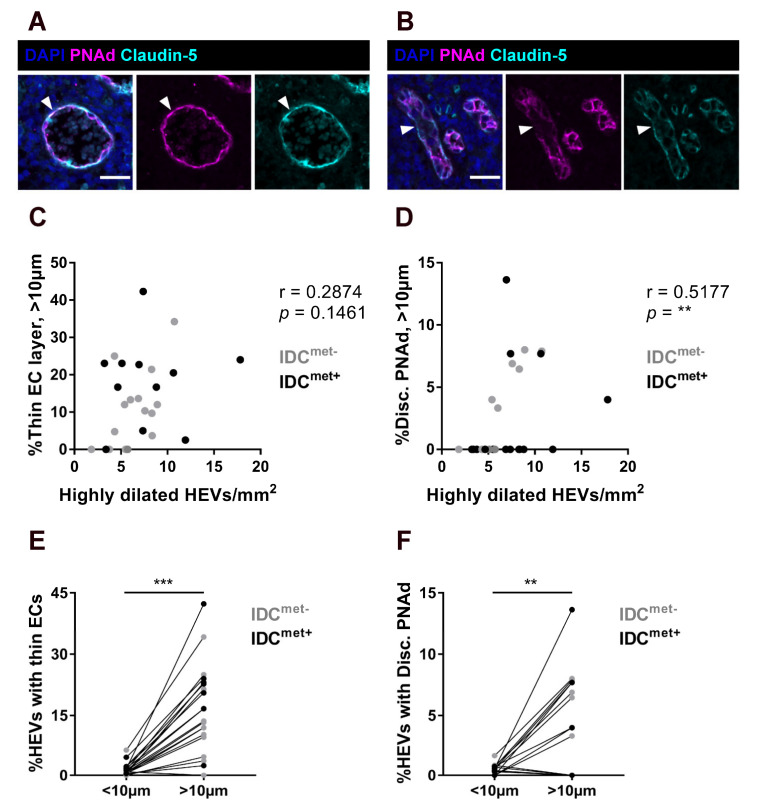
Thinning of the endothelium and discontinuous PNAd-expression in highly dilated HEVs. (**A**) Example image of an HEV (PNAd^+^/Claudin-5^+^) with thin endothelial cell (EC) layer. Arrowhead outlines thin endothelium. Scale bar: 25 µm. (**B**) Example image of an HEV with discontinuous PNAd expression. Arrowhead outlines part of the endothelium (Claudin-5^+^) with loss of PNAd (PNAd^-^). Scale bar: 25 µm (**C**) Correlation analysis of the percentage of highly dilated HEVs with thin EC layer out of all highly dilated HEVs, and the total number of highly dilated HEVs (HEVs/mm^2^) (**D**) Correlation analysis of the percentage of highly dilated HEVs with discontinuous PNAd expression out of all highly dilated HEVs, and the total number of highly dilated HEVs (HEVs/mm2). (**C,D**) n = 16 IDC^met−^ LNs from 16 patients without LN metastasis (light grey dots) and n = 11 IDC^met+^ LNs from 10 patients with LN metastasis (black dots). (**E**) Paired analysis of the frequency of thin ECs in highly dilated HEVs (>10 µm) versus HEVs with less than 10 µm dilation; n = 12 IDC^met−^ LNs from 12 patients (light grey dots) and n = 9 IDC^met+^ LNs from 8 patients (black dots). (**F**) Paired analysis of the frequency of loss of PNAd in highly dilated HEVs (>10 µm) versus HEVs with less than 10 µm dilation; n = 12 IDC^met−^ LNs from 12 patients (light grey dots) and n = 6 IDC^met+^ LNs from 5 patients (black dots). Spearman correlation test was used for correlation analysis and Paired t-test and Wilcoxon matched-pairs signed rank test were used for paired analysis. ** *p* < 0.01, *** *p* < 0.001.

Normal HEVs are characterized by their cuboidal endothelial cells (ECs) and expression of PNAd [14]. Initial examination of PNAd^+^ vessels in the LN parenchyma identified structural and molecular changes in HEV morphology that were used as the basis for the further analysis. These parameters included vessel dilation, thinning of the vessel endothelium and discontinuous PNAd-expression along the perimeter of the vessel (Figure 1A and Figure 2A,B). Dilation of HEVs was the most prominent morphological change in BC patients with IDC compared with ODs and patients with DCIS (Figure 1B). Dilated HEVs often displayed accumulation of red blood cells in the lumen, seen by autofluorescence (Figure 1A,B).

HEVs were divided into highly dilated (lumen diameter >10 µm), intermediately dilated (lumen diameter <10 µm) and non-dilated (closed lumen) (Figure 1A). Figure 1C summarizes the fraction of vessels (non-dilated, intermediately dilated and highly dilated) across all HEVs in each patient group, with an average of 10% total dilated HEVs in OD and 34% in DCIS to be compared with 48% in IDC^met−^ and 49% in IDC^met+^. Quantification of the number of analyzed vessels per mm^2^ paracortical area showed that total HEV numbers were slightly increased in patients with DCIS and IDC^met−^ compared with ODs, but with no significant differences between patients with non-invasive and invasive cancer (Figure 1D). However, major differences were observed when evaluating the presence of dilated HEVs (Figure 1E,F). While there was an increase in total number of dilated vessels in all BC patients compared to cancer-free ODs, there was a significant increase in dilated vessels in patients with IDC, also when compared with LNs from patients with DCIS (Figure 1E). No significant difference was seen in IDC patients without (IDC^met−^) compared to with LN metastasis (IDC^met+^) (Figure 1E). Also highly dilated vessels showed the strongest increase in patients with IDC, with no significant differences in patients with and without nodal metastasis (Figure 1F).

For the IDC^met−^ group, we were able to compare sentinel LNs (SLNs) with axillary LNs (ALNs) but could notably not detect a statistical difference in the total number of dilated or highly dilated vessels (Figure S2).

Taken together these data suggest that remodeling of paracortical HEVs are common pathological changes in patients with luminal invasive BC, likely to affect the whole axilla, and are independent of the presence of nodal metastasis.

### 2.2. Thinning of the Endothelium and Discontinous Expression of PNAd in Highly Dilated HEVs

We next analyzed HEVs for thinning of the vessel endothelium (Figure 2A) and discontinuous PNAd-expression (Figure 2B) in relation to vessel dilation (Figure 2C–F). There was a trend for a positive correlation with the number of highly dilated HEVs and thinning of the vessel endothelium (*p* = 0.1546) (Figure 2C), while discontinuous PNAd showed a significant positive correlation to the number of highly dilated HEVs (Figure 2D). Paired analysis, confirmed that both thinning of the endothelium (Figure 2E) and discontinuous PNAd-expression (Figure 2F) were consistently more frequent in highly dilated HEVs compared to the rest of the paracortical HEVs.

### 2.3. Partial and Complete Loss of CCL21 in Perivascular αSMA^+^ FRCs of Highly Dilated HEVs

**Figure 3:**
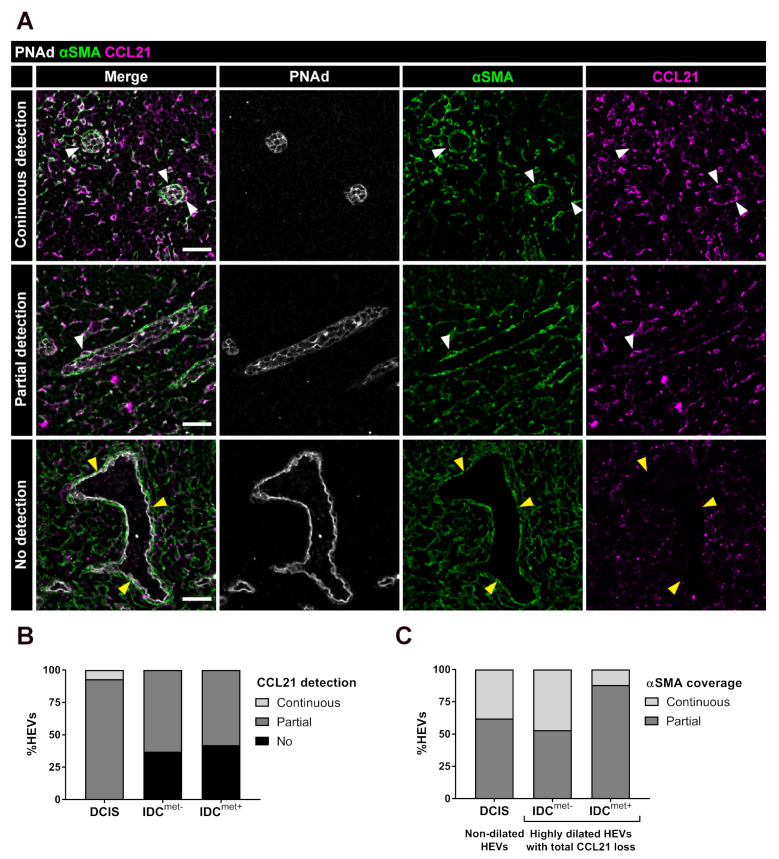
Loss of stromal CCL21 is frequent around highly dilated HEVs but is not caused by loss of perivascular αSMA^+^ cells. (**A**) Representative images of CCL21 detection in HEV-lining perivascular αSMA^+^ FRCs. LNs stained for PNAd in white outlining HEVs, αSMA in green, labelling FRCs, and the chemokine CCL21 in magenta. White arrowheads outlines FRCs with continuous or partial CCL21. Yellow arrowheads outlines highly dilated HEVs without CCL21 in perivascular αSMA^+^ cells. Examples shown from patients with DCIS (continuous and partial CCL21) and IDC^met+^ (no CCL21). Scale bar 40 µm. (**B**) Scoring of CCL21 (continuous (light grey), partial (dark grey) or no detection (black) in αSMA^+^ FRCs lining non-dilated HEVs (DCIS), and highly dilated HEVs (IDC^met−^ and IDC^met+^). (**C**) Analysis of perivascular αSMA^+^ cells lining non-dilated HEVs (DCIS) and highly dilated HEVs with no CCL21 detection (IDC^met−^ and IDC^met+^), divided in continuous coverage (light grey) or partial coverage (dark grey). (n = 5 LNs from 5 patients per group in both **B** and **C**.) The mean percentages across all patients are displayed.

The function of HEVs in recruitment of lymphocytes into the LN depends on the expression of CCL21 by surrounding perivascular and paracortical FRCs, allowing chemotaxis of CCR7^+^ naïve lymphocytes into the LN parenchyma [14]. Scoring of CCL21 around the vessel perimeter in non-dilated HEVs in LNs from patients with DCIS compared to highly dilated HEVs in patients with IDC^met−^ and IDC^met+^, revealed that a high proportion of the highly dilated HEVs in IDCs showed partial or complete loss of vessel-lining CCL21 (Figure 3A,B). Reduced staining for CCL21, was sometimes also observed in the surrounding paracortical reticular network of patients with IDC (Figure 3A, lower panel). However, paired analysis of non-dilated versus highly dilated HEVs in patients with IDC^met−^ and IDC^met+^, demonstrated that highly dilated HEVs, consistently, displayed higher frequency of CCL21 loss than non-dilated HEVs, in the same patients (Figure S3).

Loss of vessel-lining CCL21 can be either due to loss of perivascular FRCs or loss of CCL21 in the FRCs. To differentiate between these options we included staining for alpha-smooth muscle actin (αSMA) which is a common marker for FRCs and other LN mesenchymal cells [16]. Analysis of highly dilated HEVs without perivascular CCL21-detection in IDC^met−^ and IDC^met+^ LNs showed a mix of continuous and partial expression, but never a loss of αSMA^+^ cells and displayed a similar pattern of αSMA distribution as non-dilated HEVs in patients with DCIS (Figure 3C). These results support that there is a loss of CCL21 in perivascular αSMA^+^ FRCs lining highly dilated HEVs, but not a loss of αSMA^+^ perivascular cells.

### 2.4. LN Metastasis Induce Local Effects on HEVs and Perivascular FRCs

**Figure 4:**
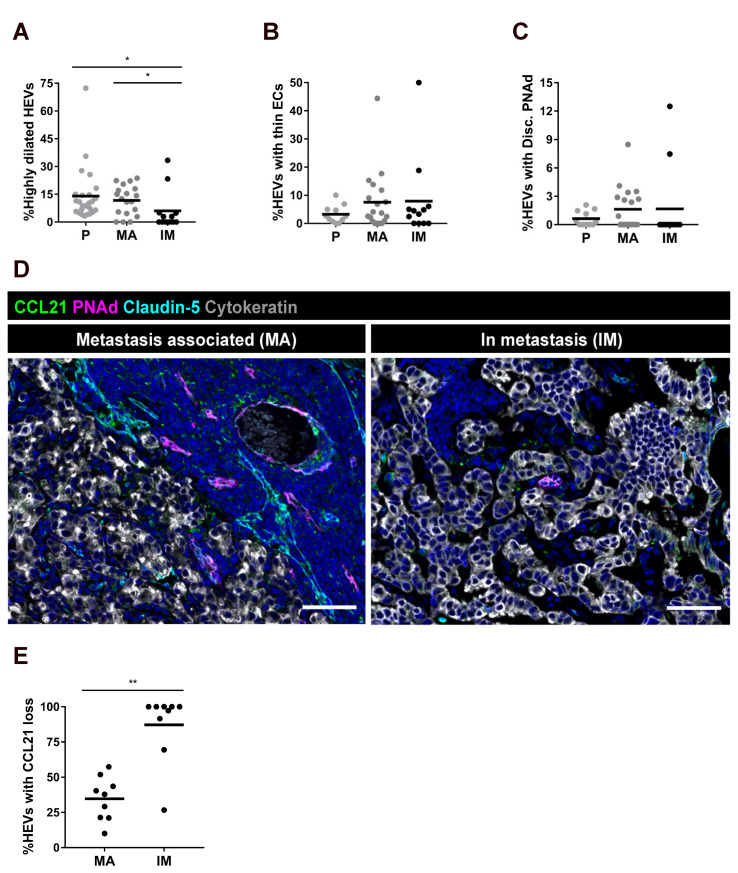
Local effects by metastatic tumor foci on HEV and FRC remodeling. (**A–C**) Groups displayed include paracortical (P), metastasis associated (MA), and in metastasis (IM)-HEVs. (**A**) Percentage of highly dilated HEVs in metastatic LNs. (**B**) Percentage of HEVs with thin endothelial cells (ECs) in metastatic LNs. (**C**) Percentage of HEVs with discontinuous PNAd expression in metastatic LNs. Solid line represent mean, dots refer to different LNs. (**D**) Representative images of LNs stained for PNAd in magenta and Claudin-5 in cyan outlining HEVs, CCL21 in green labelling FRCs and Cytokeratin in grey labelling metastatic tumor cells. Image of a MA-HEV (within 200 µm from tumor cells) and an IM-HEV within a metastasis. Scale bar 75 µm (**E**) Analysis of CCL21 loss along the perimeter of MA and IM HEVs. Dots represents analyzed LNs (n = 9 LNs from 7 patients) and the horizontal line marks the mean value. (**A–C**): Kruscal Wallis and Dunn’s multiple comparisons tests were used for statistical analysis. * *p* < 0.05; (**E**): Mann Whitney test was used for statistical analysis. ** *p* < 0.01.

In the paracortical analysis we excluded areas close to metastatic tumor cells in the IDC^met+^ group. To evaluate the potential impact of tumor metastasis on HEV remodeling, we therefore performed complementary analysis of HEVs close to metastasis (<200 µm), and within the tumor foci. While metastasis associated (MA)-HEVs displayed high dilation in a similar frequency as in the paracortex, HEVs inside metastatic foci (IM-HEVs) displayed reduced number of highly dilated vessels compared to the paracortex (Figure 4A). Vessel EC thickness and discontinuous PNAd expression did not show significant differences between the groups (Figure 4B,C).

Analysis of HEV-lining CCL21 by scoring revealed that almost all IM-HEVs lacked detectable perivascular CCL21 (87 ± 25%), and were surrounded by densely packed tumor cells (Figure 4D,E). For MA-HEVs, 35 ± 16%, had no detectable perivascular CCL21 (Figure 4D,E), thus similar as observed in the analysis of paracortical highly dilated HEV (Figure 3B, IDC^met+^, 42%). The high individual variation in all remodeling factors across the patients indicate that individual metastases induce differential effects on local HEV remodeling (Figure 4A–C,E). Perivascular αSMA^+^ cells were still present both around MA-HEVs and IM-HEVs indicating a loss of CCL21 but not loss of perivascular cells (Figure S4). Notably, metastatic burden in the analyzed sections did not correlate with paracortical HEV remodeling in patients with metastasis (Figure S5), supporting the notion that even if metastasis have local effects on LN HEV remodeling, metastasis and the widespread paracortical HEV remodeling are independent changes.

### 2.5. Accumulation of CCL21-Saturated Lymphocytes around Highly Dilated HEVs

**Figure 5:**
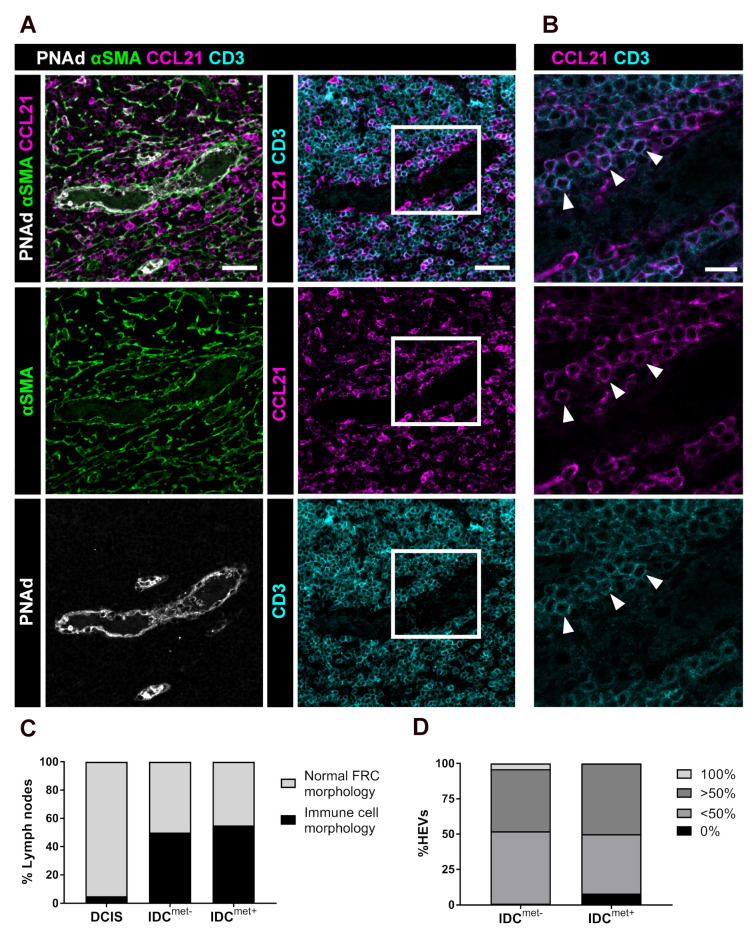
CCL21-saturated lymphocytes accumulate around dilated HEVs in patients with IDC. (**A**) TDLNs stained for PNAd in white outlining HEVs, αSMA in green labelling FRCs, CD3 in cyan (labelling T-lymphocytes) and the chemokine CCL21 in magenta. Example shown from a IDC^met−^ patient with immune cell morphology of the CCL21 staining. Scale bar 40 μm. (**B**) High magnification confocal image of the region of interest in Figure 5A. Arrowheads outlines accumulation of CD3^+^ cells with CCL21 positive staining around the vessel (immune cell morphology). Scale bar 15 µm. (**C**) Analysis of the percentage of LNs with normal FRC morphology of the CCL21 staining (CCL21^+^/αSMA^+^/CD3^−^) (light grey) or immune cell morphology (CCL21^+^/αSMA^−^/CD3^+^) (black), including DCIS (n = 19), IDC^met−^ (n = 26), IDC^met+^ (n = 31). (**D**) Quantification of CCL21 stained lymphocytes scored as: only CD3^+^ (100%) (light grey), more than 50% CD3^+^ (>50%) (dark grey), less than 50% (<50%) (medium grey) or no CD3^+^ staining (0%) (black). (n = 5 and 4 patients in IDC^met−^ and IDC^met+^ respectively.) The mean percentage across all patients is displayed.

In IDC patients, we frequently observed staining for CCL21 in αSMA^−^ cells with lymphocyte morphology (Figure 5A,B). This phenotype was common in both IDC^met−^ and IDC^met+^ patients (50% and 55% respectively) but very rare in DCIS (5%) (Figure 5C) where CCL21-expression instead was confined to αSMA^+^ FRCs (Figure S6A,B). CCL21, when bound to CCR7 is not internalized but will remain on the cell surface together with the receptor [17]. Cells that express CCR7 can therefore stain positive for CCL21. We thus hypothesized that the αSMA^−^/CCL21^+^ cells could be CCR7^+^ lymphocytes that have bound CCL21 on their surface.

To evaluate if this was the case, we stained selected LNs from the IDC^met−^ and IDC^met+^ patient groups that displayed the CCL21^+^ lymphocyte phenotype for the T-lymphocyte marker CD3 (Figure 5C). The analysis was limited to highly dilated HEVs and the CCL21^+^/αSMA^−^ cells were scored as being 100%, >50%, <50% or 0% CD3^+^ (Figure 5D). In a majority of analyzed HEVs we could confirm the presence of CCL21^+^/CD3^+^ T-lymphocytes surrounding highly dilated HEVs, often mixed with other cells with lymphocyte morphology (Figure 5D). In IDC^met+^ patients, there was a portion of HEVs where none of the CCL21^+^ lymphocyte-like cells stained positive for CD3. The latter could be explained by binding of CCL21 to incoming naïve B-lymphocytes or other CCR7^+^ immune cells. To further evaluate the presence of CCL21 on the surface of CD3^+^ T-lymphocytes we performed image analysis (Figure S7). The analysis confirms CCL21 on the surface of CD3^+^ T-lymphocytes (surface defined by CD3) in IDC with the CCL21 lymphocyte staining (Figure S7B) but not in DCIS (Figure S7A).

### 2.6. Downregulation of CCL21-Binding Heparan Sulfate

After adhesion to HEVs, lymphocyte recruitment into the LN and extravascular migration into the paracortex has been shown to be dependent on the presence of immobilized CCL21 [18]. The immobilization of CCL21 depends on the binding of the C-terminus of CCL21 to the extracellular matrix (ECM) and cell-surface sulfated glycosaminoglycans (GAGs), including heparan sulfate (HS) [19]. While human T-cells show migratory responses to immobilized CCL21 they instead arrest in response to CCL21 when the chemokine is free in solution [18].

**Figure 6:**
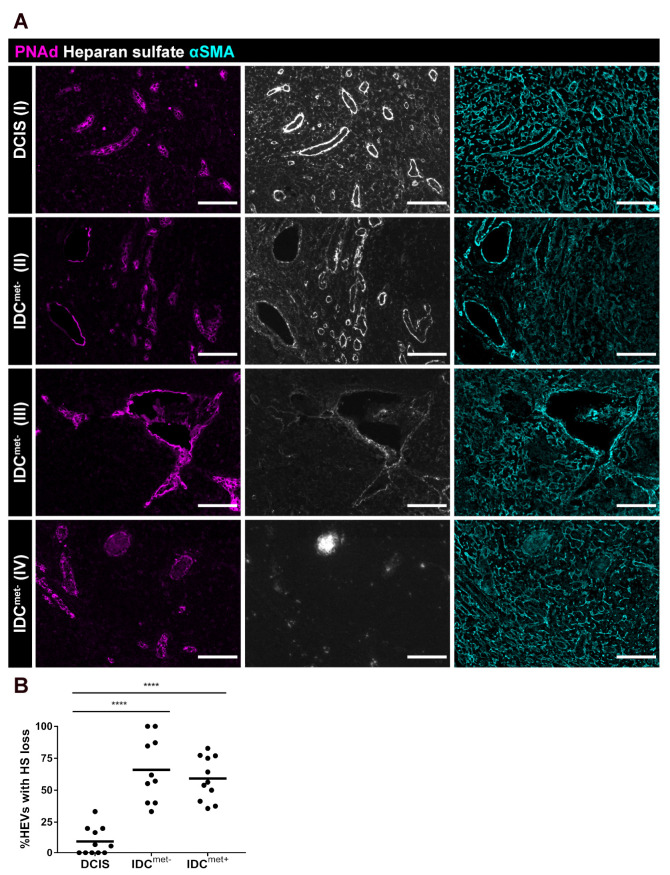
Reduced expression of stromal heparan sulfate in IDC patients. (**A**) TDLNs stained for PNAd in magenta outlining HEVs, heparan sulfate (HS) in white and αSMA in cyan, labelling FRCs. HEVs with continuous expression of HS along the vessels are shown in DCIS (A-I), HEVs with mixed continuous and loss of HS along the perimeter of the vessels are shown in IDC^met−^ (A-II), HEVs with high loss of HS along the vessel perimeter are shown in IDC^met−^ (A-III) and HEVs with total loss of HS are shown in IDC^met−^ (A-VI). Scale bar 100 µm. (**B**) Analysis of HEV-lining FRCs with loss of HS (partial or total) in LNs from DCIS (n = 11 LNs from 11 patients) and IDC^met−^ (n = 10 LNs from 10 patients), and IDC^met−^ (n = 11 LNs from 11 patients), (all IDC patients selected for analysis display accumulation of CCL21-bound lymphocytes). Dots represents analyzed LNs and the horizontal line marks the mean value. Ordinary one-way ANOVA and Holm-Sidak’s multiple comparisons test was used for statistical analysis. **** *p* < 0.0001.

To evaluate whether disruption of sulfated GAGs could be a contributing factor to the differences in CCL21 staining patterns observed in patients with IDC compared to DCIS, we stained for HS. IDC patients with the CCL21 accumulation on lymphocytes were selected for the analysis (Figure 5C). In LNs from patients with DCIS, HS staining outlined the perivascular FRCs and the reticular network of FRCs in the paracortex, which overlapped with the pattern of αSMA (Figure 6A, panel I). In patients with IDC, the HS pattern was disrupted to varying degree with a significant loss of HS around the perimeter of highly dilated HEVs in both IDC^met−^ and IDC^met+^ patients compared with non-dilated HEVs in patients with DCIS (Figure 6B). The loss of HS ranged from partial to full loss, as illustrated in images of patients with IDC^met−^ (Figure 6A, panels II-IV), to be compared with the strong continuous perivascular expression of HS in patients with DCIS (Figure 6A, panel I). Reduced staining for HS was also frequently observed in the surrounding paracortical reticular network (Figure 6A, panels III and IV), but loss of HS was notably most frequent around highly dilated HEVs compared to non-dilated HEVs in paired-analysis within selected patients with IDC, displaying high loss of HS (Figure S8). These data support that loss of HS around dilated HEVs and in the FRC network is one factor in patients with IDC that can disrupt the balance of free and immobilized cell- and matrix-bound CCL21 in the microenvironment. The latter could contribute to reduced levels of CCL21 on FRCs and to arrest of CCL21-saturated lymphocytes, binding to free CCL21 in the extracellular fluid.

## 3. Discussion

The LNs have dual roles in tumor immunology. On the one hand, LNs are critical for the induction of the anti-tumor immune response [8], on the other hand, the tumor draining LNs (TDLNs) can instead be co-opted to sustain immune tolerizing pathways and thus contribute to tumor immune escape [20,21]. LN stromal cells, which include endothelial and mesenchymal cells are vital regulators of immune cell migration and activation within the LN [6]. In this study, we address the tumor-induced effects on the LN stroma by evaluating changes to the specialized HEVs and the surrounding perivascular FRCs in axillary LNs from BC patients.

Using controlled patient groups with ER^+^, HER2^-^ luminal IDC, with and without LN metastasis, we demonstrate that LNs from patients with IDC display extensive remodeling of HEVs compared with patients with non-invasive DCIS or cancer-free ODs. Total number of HEVs did not differ between non-invasive and invasive cancer, which suggest that differences in angiogenesis is not likely to explain the differences in dysregulation of HEVs between the patient groups. It can however not be excluded as a factor behind increased total HEV numbers observed in some of the cancer-groups compared to cancer-free ODs.

Conspicuously, IDC patients with and without LN metastasis displayed remodeling of paracortical HEVs to a similar extent. Axillary and sentinel LNs in IDC patients in addition display a similar degree of HEV remodeling, indicating that the tumor-induced effects spreads across the axilla. This suggests that the widespread remodeling in the T-cell zone of the LN is induced by lymph- and/or blood-borne factors from the invasive primary tumor and not by the presence of tumor cells within the LN. Supporting this notion, we could not observe a positive correlation between the metastatic tumor cell area and the extent of paracortical HEV remodeling in IDC^met+^ patients. That vascular remodeling in TDLNs can be independent of nodal metastasis was also indicated in an earlier study by Qian et al. [12] based on 7 patients with metastatic invasive BC, where metastatic and non-metastatic LNs were compared, but from the same patients, all with LN metastasis [12]. Our data demonstrate that paracortical HEV remodeling is independent of overall nodal disease but also show that LN metastasis has local effects on HEV remodeling. This indicates that metastasis-induced LN stromal dysregulation adds to already present pre-metastatic LN stromal changes in IDC, the latter that may also facilitate metastatic seeding in the first place.

Vessel lumen dilation was linked to thinning of the EC layer and less frequently also to discontinuous PNAd-expression along the vessel perimeter. The plump morphology of HEVs has been shown to at least partly depend on accumulation of migrating lymphocytes forming so called HEV “pockets” in association with the EC [22] and blockage of lymphocyte recruitment through anti-L-selectin antibodies, caused thinning of the HEV endothelium in mice [22]. Thus, thinning of the endothelium may in itself be a sign of reduced lymphocyte recruitment across the vessel. Lymphocyte distribution and interaction with EC and stroma in association to dilated HEVs would be of interest to evaluate in follow up studies designed for electron microscopy.

Beside endothelial changes, dilated HEVs displayed dysregulation of perivascular FRCs that included loss of CCL21 and reduced levels of CCL21-binding heparan sulfate (HS), but not loss of perivascular αSMA^+^ cells. Downregulation of CCL21-expression has been reported in TDLNs in mouse models of melanoma, both in FRCs [23] and in HEVs [24]. Mouse HEV EC in contrast to human HEV ECs, directly express CCL21 [25]. Also single cell transcriptional data from human BC support that transcriptional downregulation of CCL21 in FRCs in sentinel LNs, can occur [26].

The changes of perivascular FRCs reported here (i.e., loss of CCL21 and HS) were consistently more frequent around highly dilated HEVs, when compared to non-dilated HEVs, in the same patients. This support that in human invasive BC, remodeling of the HEVs is associated with concomitant dysregulation of perivascular FRCs. Less frequent perivascular changes could also be observed around non-dilated HEVs in IDC and it is possible that dysfunction of perivascular FRCs precedes HEV remodeling. Notably, downregulation of CCL21 detection in perivascular FRCs lining dilated HEVs could be observed together with (Figure 5A), but also without (Figure 3A, lower panel), accumulation of CCL21-saturated lymphocytes, discussed below.

The immobilization of CCL21 to the ECM is necessary for efficient T-cell migration in the LN parenchyma [18] and while human naïve CCR7^+^ T-lymphocytes show migration in response to immobilized CCL21 they instead arrest in response to CCL21 in solution [18]. In experimental settings, pre-incubating T-lymphocytes with CCL21 can also interfere with LN homing [27]. As CCL21, when bound to CCR7 is not internalized but will remain on the cell surface together with the receptor [17], CCR7 positive cells can stain positive for CCL21 [17]. Thus, changes in the balance of immobilized CCL21 to free CCL21 in solution is expected to inhibit effective migration of lymphocytes from the HEVs into the LN parenchyma and to lead to increased accumulation of CCL21 on the surface of arrested CCR7^+^ cells. The high numbers of CCL21-surface bound lymphocytes close to HEVs in patients with IDC, demonstrated here, that was linked to concomitant loss of HS, supports this model, a phenomenon that was very rare in patients with non-invasive DCIS (1/19 analyzed LNs). Experimental models able to mimic HEV dilation and loss of perivascular HS would be required to evaluate this model. Besides effects on HS, changes in other CCL21 binding sulfated GAGs, such as chondroitin sulfate [28], or increased C-terminal processing of the long GAG-binding form of CCL21, into the truncated soluble CCL21 [19], may also contribute to increased levels of CCL21 in solution.

Changes in expression of sulfated protein glycans (PG) in the primary tumor is frequently reported and can have multiple effects on e.g., tumor invasion, survival, proliferation and tumor immune evasion [29]. Pre-metastatic effects caused by changes in HS has however, to our knowledge, not earlier been described. Loss of HS may not only influence chemokine presentation in the TDLNs but could have multiple effects since HSPGs interact with many different proteins including growth factors and structural proteins [29,30]. Changes in HS can be caused by changes in expression of the cell surface HSPG syndecans and glypicans, changes in expression of ECM HSPG perlecan agrin and collagen XVIII, changes in the levels of HS biosynthesis or in expression of heparanase enzymes able to degrade HS chains in the ECM [30]. The underlying mechanism for downregulation of HS and dysregulation of FRC functions in TDLNs in IDC could be multifactorial and will be an interesting question for continued research.

TDLNs have potential as therapeutic targets in immunotherapy [21]. To understand how invasive cancer modulate the LN immune function will thus be of value to find new ways to unleash suppression of anti-tumor responses. In this context, both the underlying mechanisms to HEV and FRC dysregulation and the consequences of LN HEV remodeling for tumor immunity still needs to be further explored. This will require innovative strategies to study the vasculature and stroma in human TDLN.

Lymphotoxin beta receptor (LTBR) signaling is required for maintaining HEV functions in mice [31] and endothelial-specific deletion of LTBR results in reduced LN formation with effects on HEVs in the LNs that do form [32]. HEV function is also lost in LNs if the influx of lymph is blocked in mouse models [33,34] with downregulation of HEV marker expression, including reduced PNAd expression, and thinning of the vessel. Similar effects are seen if dendritic cells (DCs) are depleted [35]. Evidence support that mouse HEV markers and glycotopes are maintained, at least partly, through LTBR signaling where DCs can provide LT-α, LT-β and LIGHT, ligands for LTBR [35]. Interestingly, in neither of these mouse models, dilation of HEVs was reported. In our cohort of patients with IDC, dilation of the vessels is the major effect and thinning of the vessel EC layer or discontinuous PNAd expression is rarely seen without concomitant vessel dilation, with exception for HEVs inside tumor foci. Changes in DC distribution within BC sentinel LNs compared with LNs from healthy controls has been reported [36], but it remains to be established if it is also a contributing factor to HEV remodeling in human TDLNs.

The variation seen between different IDC patients with more or less severe HEV remodeling and with different degree of stromal functional disruption, with and without accumulation of CCL21 on lymphocytes, is likely to influence their ability to induce effective anti-tumor responses. It is tempting to speculate that defects in T- and B-lymphocyte recruitment, spurred by the dysregulation of both HEVs and FRC functions, will contribute to a reduced lymphocyte clonal availability. Disruption of paracortical immobilized CCL21-gradients is also likely to reduce the possibilities for effective interactions between T-lymphocytes and DCs. Our study was not designed to evaluate this, but lay a foundation for continued research. New data do support immune escape in the transition from DCIS to IDC, where interestingly T-lymphocyte receptor clonotype diversity was significantly higher in DCIS than in IDCs, in analysis of primary tumors [37]. This would be in line with our findings, where patients with DCIS also show less or no effects on HEV dilation and where disruption of LN stromal CCL21 or HS are very rare. High proportion of CD3 (T-lymphocytes) and CD20 (B-lymphocytes) in the TDLNs has interestingly also been associated with prolonged disease-free survival in BC [38], which could reflect protective effects of retained ability to recruit lymphocytes.

Taken together, we show a first example of how stromal and vascular dysregulation in TDLNs reflect the primary tumor invasiveness and differentiate invasive from non-invasive cancer. The changes were present both in pre-metastatic and metastatic LNs, across both sentinel and non-sentinel axillary nodes. Unexpectedly, changes in HEVs and HEV-lining FRCs were associated with accumulation of CCL21-saturated lymphocytes in a large fraction of the patients and observed together with loss of perivascular HS. Future studies are warranted to expand our findings to different subtypes of BC and additional human cancer forms and to evaluate LN vascular and stromal changes as biomarkers. To this end artificial intelligence (AI)-based image analysis methods for identification of changes in HEV morphology would be an important next step for allowing high throughput analysis of large cohorts of patients.

## 4. Materials and Methods

### 4.1. Biobank Material and Ethical Considerations

Formalin fixed and paraffin embedded (FFPE) biobank LNs from patients with DCIS (n = 19 LNs from 19 patients); non-invasive, non-metastatic BC, and IDC, with (IDC^met+^, n = 33 LNs from 29 patients) and without (IDC^met−^, n = 26 LNs from 26 patients) LN metastasis were used for immunostaining (Table S1). The number of patients and LNs used in individual analyses are specified below and in each figure legend. The article is based on biobank material. In Sweden there is a Biobanks in Medical Care Act (SFS 2002:297), later complemented with Regulation (“Förordning”) (SFS 2002:746) regarding biobanks in areas such as health and medical services. The law applies to the biobanks that consist of tissue samples taken and collected for a specific purpose from patients or other donors within healthcare. Pancreatic LNs from cancer-free organ donors (ODs) (n = 15 LNs from 10 ODs) were collected separately from anonymized donors and were used as controls for HEV morphology and density. The ethical permit allows for analyzing anonymized coded samples from the Uppsala biobank without consent from individual patients and from OD. The work has gone through careful ethical evaluation by the regional ethical committee in Uppsala Sweden. Approval: 2017/061 and addition 2017/061:1 and 2017/061:2. The study was conducted in accordance with the Helsinki Declaration of 1975, revised in 1983.

### 4.2. Immunofluorescence Staining

Four µm FFPE sections were baked for 1 h at 60 °C, deparaffinized and rehydrated. Antigens were retrieved by incubation in 1 mM ethylenediaminetetraacetic acid (EDTA) (Invitrogen, Carlsbad, CA, USA) (pH 9) or 10 mM sodium citrate buffer (pH 6) (Table S2). The sections were blocked with 0.033% streptavidin in phosphate buffered saline (PBS) with 0.05% Tween20 (PBST) for 15 min, 0.0033% biotin in PBST and 5% donkey serum in PBST (blocking buffer) at RT (all reagents Sigma-Aldrich, St. Louis, MO, USA). After blocking, sections were incubated with primary antibodies (Table S2) overnight followed by washing in PBST and incubation with polyclonal secondary donkey antibodies (Table S3) for 30 min. For biotin-streptavidin amplification of PNAd detection, the sections incubated with CF647 conjugated streptavidin (1:500) (Biotium, Hayward, CA, USA) for 30 min.

Staining for heparan sulfate was performed in two steps using tyramide signal amplification (TSA) kit (Perkin Elmer, Waltham, MA, USA) followed by antibody stripping, allowing detection of two antibodies of the same serotype. Briefly, antigens were retrieved and the sections were blocked, incubated with primary antibodies overnight and biotinylated secondary antibodies for 30 min (Tables S2 and S3), as described above. Thereafter the sections incubated with HRP-conjugated streptavidin (1:500) followed by TSA-Cy5 (1:150) (Perkin Elmer). Antibody complexes were stripped by antigen retrieval, as described above. The sections were again blocked and incubated with primary antibodies for 1 h at RT, followed by secondary antibodies for 30 min, and CF594 conjugated streptavidin (1:500) (Biotium) for 30 min.

In all stainings, sections were counterstained with 4′,6-Diamidine-2′-phenylindole dihydrochloride (DAPI) (Invitrogen) and mounted with #1.5 coverslips and ProLong Gold antifade mounting media (Molecular Probes, Eugene, OR, USA).

### 4.3. Imaging

Images were acquired using Vectra Polaris™ Automated Quantitative Pathology Imaging System (Akoya Biosciences, Menlo Park, CA, USA). For the images in Figure 5B, Figures S6B and S7, images were acquired with Leica TCS SP8 confocal microscope with HC PL APO CS2 63x/1.30 GLYC objective (Leica, Wetzlar, Germany).

### 4.4. Image Analysis

RGB (red green blue) snapshots were acquired in Phenochart 1.0.12 (Akoya Biosciences). Image analysis was performed using the open source program Fiji ImageJ version 1.52p.

#### 4.4.1. Vessel Morphology

HEVs in the paracortex were counted manually and classified as non-dilated, if the lumen was closed, intermediately dilated; if the mean of the shortest lumen diameter (three measurements per vessel of the shortest cross-sectional diameter) was smaller than 10 µm, and highly dilated; if the mean was larger than 10 µm (Figure 1A and Figure S1). Classification also included lack of the cuboidal shaped endothelium (thin endothelial cell (EC) layer), and discontinuous PNAd expression (Figure 2A,B). An average of 3.5 mm^2^ ± 0.8 mm^2^ of the paracortex (defined as CCL21^+^ area >200 µm from the tumor border and from parenchymal fat) and 255 ± 99 HEVs per LN were analyzed. The analysis included OD (n = 15 LNs from 10 patients), DCIS (n = 19 LNs from 19 patients), IDC^met−^ (n = 25 LNs from 25 patients) and IDC^met+^ (n = 25 LNs from 24 patients).

#### 4.4.2. CCL21-Expression and Perivascular αSMA^+^ Cells

Paracortical HEVs of DCIS (non-dilated) and IDC^met−^ and IDC^met+^ (highly dilated) were selected for analysis (n = 5 patients per group, n = 19 ± 2 analyzed HEVs per patient). CCL21 was categorized as continuous, partial, or no detection. To determine the presence of perivascular αSMA^+^ cells lining vessels with different CCL21-status, HEVs with continuous or partial CCL21 were selected from DCIS and HEVs with no detection were selected from IDC^met−^ and IDC^met+^. The vessels were categorized as having continuous, partial, or no coverage with αSMA^+^ cells.

In addition the frequency of CCL21 loss in αSMA^+^ cells lining non-dilated versus highly dilated HEVs of IDC^met−^ and IDC^met+^ was determined (n = 5 patients per group, n = 20 ± 9 analyzed non-dilated HEVs per patient versus n = 21 ± 9 highly-dilated).

#### 4.4.3. Metastasis Associated (MA) and in Metastasis (IM) HEVs

MA-HEVs (within 200 µm from the metastatic border) and IM-HEVs (within the metastasis) were counted and the percentages of highly dilated, thin EC layer and HEVs with discontinuous PNAd expression were calculated (n = 19 LNs from 16 patients with n = 67 ± 53 MA-HEVs per patient, and n = 12 LNs from 10 patients with n = 21 ± 20 IM-HEVs per patient). The status of CCL21 in the HEV-lining FRCs was characterized as continuous, partial, or no detection (n = 9 LNs from seven patients with n = 78 ± 62 MA-HEVs per patient and n = 22 ± 4 IM-HEVs per patient).

#### 4.4.4. Metastatic Burden

The area of the metastasis in IDC^met+^ LNs (n = 18 LNs from 17 patients) was calculated as a percentage of the total area of the LN. A region of interest was made based on the DAPI staining and a threshold of 6–255 (min-max) was set for the measurement or the total LN area. Secondary regions of interest were made for the individual metastatic foci using the cytokeratin staining and a threshold of 35–255 (min-max) was used to measure the area occupied by the tumor cells.

#### 4.4.5. CCL21^+^ Immune Cells

The morphology (reticular or immune cell) of the CCL21 staining was examined in all LNs from all patient groups. Five LNs from IDC^met−^ and four LNs from IDC^met+^ which displayed immune cell morphology were selected and stained for CD3 to determine the origin of the CCL21^+^ cells with immune cell morphology. Highly dilated HEVs from IDC^met−^ and IDC^met+^ (n = 14 ± 6 HEVs per patient) were selected and scored as having only (100%), more than 50% (>50%), less than 50% (<50%), or no (0%) CD3^+^/CCL21^+^/αSMA^−^ cells. For the localization analysis in Figure S7 the “Plot Profile” tool was used in Fiji Image J.

#### 4.4.6. Heparan Sulfate

Heparan sulfate (HS) expression in perivascular αSMA^+^ cells was analyzed in non-dilated paracortical HEVs in LNs with reticular CCL21 morphology from DCIS patients (n = 11 LNs from 11 patients, n = 11 ± 4 HEVs per patient) and highly dilated paracortical HEVs from IDC^met−^ (n = 10 LNs from 10 patients, n = 16 ± 14 HEVs per patient) and IDC^met+^ (n = 11 LNs from 11 patients, n = 28 ± 13 HEVs per patient) with immune cell CCL21 morphology. The vessels were categorized as having continuous HS-expression or full or partial loss of HS lining the vessel perimeter.

In addition the frequency of HS loss in αSMA^+^ cells lining non-dilated versus highly dilated HEVs of IDC^met−^ and IDC^met+^ was determined (n = 5 patients per group, n = 15 ± 5 analyzed non-dilated HEVs per patient versus n = 18 ± 11 highly-dilated).

### 4.5. Statistical Analysis

Statistical analysis was performed with GraphPad Prism Software (GraphPad Prism, Software, San Diego, CA, USA). To determine the distribution of each data set D’Agostino and Pearson, Shapiro-Wilk and KS normality tests were used. In the case of normal distribution, parametric analyses were performed with ordinary one-way ANOVA and Holm-Sidak’s multiple comparison test, paired t-test, unpaired t-test and Spearman correlation test. In the case of non-normal distribution non-parametric analyses were performed using Kruscal-Wallis test and Dunn’s multiple comparison test, Wilcoxon matched-pairs signed rank test and Mann Whitney test were used. The use of each test is specified in the figure legends.

## 5. Conclusions

We present a first example of how HEV and perivascular stromal cell dysregulation in human TDLNs reflect the primary tumor invasiveness and differentiates invasive from non-invasive BC. The main changes were pre-metastatic, and our data support that LN metastasis adds to already present dysregulation of LN functions. The immunological consequences of the HEV remodeling included a perivascular-accumulation of CCL21-saturated lymphocytes, in a large fraction of the patients with IDC, which we link to loss of perivascular HS in FRCs. Pre-metastatic tumor-induced dysregulation of GAGs, such as HS, is still an unexplored area. We present multicolor antibody panels for FFPE biobank samples, used in regular pathological analysis, which will allow both future evaluation of HEV and stromal changes as biomarkers and facilitate the analysis of other tumor types.

## Data Availability

The data presented in this study are available on request from the corresponding author.

## Supplementary Materials

The following are available online at https://www.mdpi.com/2072-6694/13/2/211/s1, Figure S1: Method for image analysis.; Figure S2: Similar degree of HEV dilation in non-metastatic sentinel and axillary LNs draining IDC.; Figure S3: Loss of CCL21 in highly dilated HEVs; Figure S4: αSMA staining of MA-HEVs and IM-HEVs. Figure S5: Correlation analysis of metastatic burden and paracortical remodeling of HEVs; Figure S6: CCL21 is confined to HEV-lining FRCs around non-dilated HEVs; Figure S7: Image analysis of CCL21 and CD3 in DCIS and IDC; Figure S8: Loss of HS in highly dilated HEVs; Table S1: Clinical data of the patient cohort; Table S2: Primary antibodies; Table S3: Secondary antibodies.
