# Supplementary material for: Remodeling of the Lymph Node High Endothelial Venules Reflects Tumor Invasiveness in Breast Cancer and is Associated with Dysregulation of Perivascular Stromal Cells
Source: Cancers (Basel). 2021 Jan 8;13(2):211. doi: 10.3390/cancers13020211 (PMC7827313; doi:10.3390/cancers13020211)
Supplement: Supplementary file 1 [file cancers-13-00211-s001.zip › Supplementary Data/Supplementary Table 1.docx]

**Table S1: Clinical data of the patient cohort.** Clinical data for patients with ductal carcinoma in situ (DCIS), invasive ductal carcinoma (IDC) with (IDC^met+^) and without (IDC^met−^) LN metastasis. The data include age at surgery, the expression status of the primary tumor: estrogen receptor (ER), progesterone receptor (PR), human epidermal growth factor receptor 2 (HER2), and the status of disease recurrence and metastasis. The expression pattern of the primary tumor is only evaluated for IDC tumors and is therefore non applicable (na) for DCIS.

| Patient: | Age at surgery: | ER: | PR: | HER2: | Disease recurrence and metastasis: |
| --- | --- | --- | --- | --- | --- |
| DCIS1 | 62 | na | na | na | No new relevant clinical data. |
| DCIS2 | 63 | na | na | na | Local metastasis in the breast. |
| DCIS3 | 59 | na | na | na | No new relevant clinical data. |
| DCIS4 | 49 | na | na | na | No new relevant clinical data. |
| DCIS5 | 64 | na | na | na | No new relevant clinical data. |
| DCIS6 | 59 | na | na | na | No new relevant clinical data. |
| DCIS7 | 62 | na | na | na | No new relevant clinical data. |
| DCIS8 | 62 | na | na | na | No new relevant clinical data. |
| DCIS9 | 55 | na | na | na | No new relevant clinical data. |
| DCIS10 | 73 | na | na | na | No new relevant clinical data. |
| DCIS11 | 48 | na | na | na | No new relevant clinical data. |
| DCIS12 | 68 | na | na | na | No new relevant clinical data. |
| DCIS13 | 71 | na | na | na | No new relevant clinical data. |
| DCIS14 | 55 | na | na | na | No new relevant clinical data. |
| DCIS15 | 66 | na | na | na | New DCIS. Contralateral IDC. |
| DCIS16 | 72 | na | na | na | No new relevant clinical data. |
| DCIS17 | 73 | na | na | na | No new relevant clinical data. |
| DCIS18 | 67 | na | na | na | No new relevant clinical data. |
| DCIS19 | 78 | na | na | na | No new relevant clinical data. |
| Patient: | **Age at surgery:** | **ER:** | **PR:** | **HER2:** | **Clinical follow up:** |
| IDC^met−^1 | 60 | + | + | − | No new relevant clinical data. |
| IDC^met−^2 | 54 | + | + | − | Lipoma in the same breast. |
| IDC^met−^3 | 52 | + | + | − | No new relevant clinical data. |
| IDC^met−^4 | 62 | + | − | − | No new relevant clinical data. |
| IDC^met−^5 | 62 | + | + | − | No new relevant clinical data. |
| IDC^met−^6 | 62 | + | + | − | No new relevant clinical data. |
| IDC^met−^7 | 68 | + | + | − | Distant metastasis. |
| IDC^met−^8 | 59 | + | − | − | No new relevant clinical data. |
| IDC^met−^9 | 51 | + | 0.05 | − | No new relevant clinical data. |
| IDC^met−^10 | 82 | + | + | − | No new relevant clinical data. |
| IDC^met−^11 | 73 | + | + | − | No new relevant clinical data. |
| IDC^met−^12 | 71 | + | + | − | No new relevant clinical data. |
| IDC^met−^13 | 52 | + | + | − | No new relevant clinical data. |
| IDC^met−^14 | 87 | + | + | − | No new relevant clinical data. |
| IDC^met−^15 | 58 | + | + | − | No new relevant clinical data. |
| IDC^met−^16 | 78 | + | + | − | Contralateral benign intraductal papilloma. |
| IDC^met−^17 | 53 | + | − | − | No new relevant clinical data. |
| IDC^met−^18 | 56 | + | + | − | No new relevant clinical data. |
| IDC^met−^19 | 66 | + | + | − | No new relevant clinical data. |
| IDC^met−^20 | 58 | + | + | − | No new relevant clinical data. |
| IDC^met−^21 | 32 | + | + | − | No new relevant clinical data. |
| IDC^met−^22 | 68 | + | + | − | No new relevant clinical data. |
| IDC^met−^23 | 43 | + | + | − | No new relevant clinical data. |
| IDC^met−^24 | 81 | + | + | − | No new relevant clinical data. |
| IDC^met−^25 | 76 | + | + | − | No new relevant clinical data. |
| IDC^met−^26 | 69 | + | + | − | No new relevant clinical data. |
| Patient: | **Age at surgery:** | **ER:** | **PR:** | **HER2:** | **Clinical follow up:** |
| IDC^met+^1 | 53 | + | + | − | No new relevant clinical data. |
| IDC^met+^2 | 67 | + | + | − | Distant metastases. |
| IDC^met+^3 | 64 | + | + | − | No new relevant clinical data. |
| IDC^met+^4 | 68 | + | + | − | No new relevant clinical data. |
| IDC^met+^5 | 77 | + | + | − | No new relevant clinical data. |
| IDC^met+^6 | 46 | + | + | − | Distant metastasis. Recurrence in breast.  Further distant metastases. |
| IDC^met+^7 | 59 | + | + | − | No new relevant clinical data. |
| IDC^met+^8 | 87 | + | + | − | No new relevant clinical data. |
| IDC^met+^9 | 65 | + | + | − | No new relevant clinical data. |
| IDC^met+^10 | 64 | + | + | − | No new relevant clinical data. |
| IDC^met+^11 | 64 | + | + | − | Distant metastasis from the IDC. |
| IDC^met+^12 | 52 | + | + | − | Multiple distant metastases. |
| IDC^met+^13 | 53 | + | + | − | No new relevant clinical data. |
| IDC^met+^14 | 63 | + | + | − | Distant metastases and contralateral LN metastasis. |
| IDC^met+^15 | 60 | + | + | − | No new relevant clinical data. |
| IDC^met+^16 | 55 | + | 0.05 | − | No new relevant clinical data. |
| IDC^met+^17 | 60 | + | + | − | No new relevant clinical data. |
| IDC^met+^18 | 68 | + | + | − | No new relevant clinical data. |
| IDC^met+^19 | 61 | + | + | − | No new relevant clinical data. |
| IDC^met+^20 | 59 | + | + | − | Contralateral IDC without metastasis. |
| IDC^met+^21 | 53 | + | + | − | No new relevant clinical data. |
| IDC^met+^22 | 37 | + | + | − | No new relevant clinical data. |
| IDC^met+^23 | 44 | + | + | − | No new relevant clinical data. |
| IDC^met+^24 | 50 | + | + | − | No new relevant clinical data. |
| IDC^met+^25 | 83 | + | + | − | No new relevant clinical data. |
| IDC^met+^26 | 85 | + | + | − | No new relevant clinical data. |
| IDC^met+^27 | 63 | + | + | − | No new relevant clinical data. |
| IDC^met+^28 | 66 | + | + | − | No new relevant clinical data. |
| IDC^met+^29 | 59 | + | + | − | No new relevant clinical data. |
