# Supplementary material for: Remodeling of the Lymph Node High Endothelial Venules Reflects Tumor Invasiveness in Breast Cancer and is Associated with Dysregulation of Perivascular Stromal Cells
Source: Cancers (Basel). 2021 Jan 8;13(2):211. doi: 10.3390/cancers13020211 (PMC7827313; doi:10.3390/cancers13020211)
Supplement: Supplementary file 1 [file cancers-13-00211-s001.zip › Supplementary Data/Supplementary Table 2.docx]

| **Antigen:** | **Host:** | **Class:** | **Clone:** | **Catalogue nr:** | **Company:** | **Dilution:** | **Retrieval:** |
| --- | --- | --- | --- | --- | --- | --- | --- |
| CCL21 | Goat | Polyclonal | na | AF366 | R&D Systems, | 1:20 | pH 9 |
| CD3e | Rabbit | Monoclonal | 1H4 | ZRB1011 | Sigma Aldrich | 1:100 | pH 9 |
| Claudin-5 | Rabbit | Monoclonal | EPR7583 | Ab131259 | Abcam | 1:100 | pH 9 |
| Cytokeratin | Mouse | Monoclonal | MNF116 | M0821 | Dako | 1:100 | pH 9 |
| Cytokeratin 18 | Rabbit | Polyclonal | na | PA5-14263 | Invitrogen | 1:20 | pH 9 |
| Pan cytokeratin | Rabbit | Polyclonal | na | NB600-579 | Novusbio | 1:20 | pH 9 |
| Heparan sulfate | Mouse | Monoclonal | F58-10E4 | 370255-1 | Amsbio | 1:20 | pH 6 |
| PNAd | Rat | Monoclonal | MECA-79 | 553863 | BD Pharmingen | 1:20 | pH 9 |
| αSMA | Mouse | Monoclonal | 1A4 | M085129-2 | Dako | 1:20 | pH 9 |

**Table S2: Primary antibodies.** Table of primary antibodies used in the immunofluorescence stainings. Including information of the antigen, host, class, clone, catalogue number, company, dilution and retrieval solution. (na: non applicable)
