# Supplementary material for: Remodeling of the Lymph Node High Endothelial Venules Reflects Tumor Invasiveness in Breast Cancer and is Associated with Dysregulation of Perivascular Stromal Cells
Source: Cancers (Basel). 2021 Jan 8;13(2):211. doi: 10.3390/cancers13020211 (PMC7827313; doi:10.3390/cancers13020211)
Supplement: Supplementary file 1 [file cancers-13-00211-s001.zip › Supplementary Data/Supplementary Table 3.docx]

| **Species Reactivity:** | **Conjugate:** | **Catalogue nr:** | **Company:** | **Dilution:** |
| --- | --- | --- | --- | --- |
| Goat | AF488 | A110559 | Invitrogen | 1:300 |
| Goat | AF555 | A2142 | Invitrogen | 1:300 |
| Mouse | AF488 | 715-545-150 | Jackson ImmunoResearch | 1:300 |
| Mouse | AF594 | A21203 | Invitrogen | 1:300 |
| Mouse | Biotin | 715-065-151 | Jackson ImmunoResearch | 1:200 |
| Mouse | Cy3 | 715-165-151 | Jackson ImmunoResearch | 1:300 |
| Rabbit | AF555 | A31572 | Invitrogen | 1:300 |
| Rabbit | AF594 | A21207 | Invitrogen | 1:300 |
| Rat | Biotin | 712-065-153 | Jackson ImmunoResearch | 1:200 |

**Table S3: Secondary antibodies.** Table of secondary antibodies used in the immunofluorescence stainings. Including information of species reactivity, conjugate, catalogue number (nr), company, and dilution.
